# Excess Weight May Account for More Than 10% of All Cancers: The Underestimated Impact of the Obesity Epidemic

**DOI:** 10.34133/cancomm.0040

**Published:** 2026-07-02

**Authors:** Luna Kiran Adhikari, Fatemeh Safizadeh, Marko Mandic, Hermann Brenner

**Affiliations:** ^1^ Division of Clinical Epidemiology of Early Cancer Detection, German Cancer Research Centre (DKFZ), Heidelberg, Germany.; ^2^ Institute for Medical Information Processing, Biometry and Epidemiology (IBE), Faculty of Medicine, LMU Munich, Munich, Germany.; ^3^ Pettenkofer School of Public Health, Munich, Germany.; ^4^ Cancer Prevention Graduate School, German Cancer Research Centre (DKFZ), Heidelberg, Germany.

The proportion of all cancers attributable to excess weight has been estimated at 2% to 8%, with higher population attributable fractions (PAFs) for women than for men [1–3]. However, this burden may be underestimated. Most prior estimates relied on body mass index (BMI), which may not adequately capture adiposity-related cancer burden. Standard BMI categories do not account for variation in body composition by age, sex, race, ethnicity, or metabolic status, increasing the risk of misclassification [4]. BMI does not distinguish between fat and lean mass, nor does it reflect fat distribution, which is more closely associated with metabolic risk [5]. In contrast, measures of central adiposity, such as waist–hip ratio (WHR) or waist circumference (WC), may better predict cancer risk. These measures more accurately reflect visceral fat, which is strongly associated with metabolic dysfunction [6,7].

Estimates may also be influenced by risk below conventional adiposity thresholds and by prediagnostic weight loss, whereby undiagnosed cancer leads to cachexia-related weight loss prior to diagnosis and weakens observed associations [8]. Few studies have examined how these methodological factors jointly influence estimates of the population burden of cancer. In this study, we assessed how these factors jointly influence estimates of the cancer burden attributable to excess weight. While adiposity-related associations are established for specific cancer sites, we focused on overall cancer burden, aiming to provide a more comprehensive estimate that also captures potential contributions to cancers, including rarer types, for which evidence may still be limited or evolving. Using UK Biobank (UKB) data, we compared PAFs derived from BMI, WC, and WHR, evaluated quartile-based versus standard classifications based on National Health Survey England (NHS) cutoffs (Table S1), and assessed the impact of excluding early follow-up to reduce reverse causation. Hazard ratios (HRs) were estimated using multivariable Cox models, and PAFs were calculated with corresponding 95% confidence intervals. Models using Fine and Gray models yielded similar results (Table S2); therefore, Cox models were retained for primary analyses. Methods are detailed in the Supplementary Materials.

Of the 502,422 UKB participants, we excluded those with prior cancer (except non-melanoma skin cancer), withdrawn consent, or missing exposure data, leaving 458,543 participants (Fig. S1). During a median follow-up of 11.8 (interquartile range [IQR], 10.9 to 12.5) years, 50,136 participants were diagnosed with cancer. At baseline, the median age was 57 (IQR, 50 to 63) years, and 53.3% were women. The median BMI was 26.7 kg/m^2^, the median WC was 83.0 cm in women and 96.0 cm in men, and the median WHR was 0.81 and 0.93, respectively (Table S3).

Table 1 compares associations of adiposity measures with overall cancer risk. Quartile-based classifications yielded stronger associations, particularly for WC and WHR. After excluding the first 4 years of follow-up, HRs and PAFs increased for all measures. Restricting the analysis to the 7- to 14-year follow-up period, quartile-based PAFs exceeded standard estimates by +0.4 (BMI), +6.8 (WC), and +3.6 (WHR) percentage points. PAF estimates more than doubled from 5.5% for BMI standard categorization and no exclusion, to 11.5% for derivation from WC quartiles, excluding the first 7 years (Table S4). This difference reflects the combined influence of the adiposity measure used, the exposure categorization approach, and the exclusion of early follow-up.

**Table 1.** Comparison of associations of adiposity measures with cancer risk and of PAFs across follow-up periods.

Follow-up period, years	No. of cancer^a^ cases	Hazard ratio (95% CI)					
		Categories	BMI^b^	Categories	Waist circumference^c^	Categories	Waist–hip ratio^d^
0–14	50,136	*Normal*	*Reference*	*Normal*	*Reference*	*Normal*	*Reference*
		*Overweight*	*1.05 (1.03–1.08)*	*Increased*	*1.13 (1.11–1.15)*	*Increased*	*1.12 (1.10–1.15)*
		*Obese*	*1.14 (1.11–1.17)*	*NA*	*NA*	*NA*	*NA*
		*PAF (%, 95% CI)*	*5.5 (3.9–6.9)*	*PAF (%, 95% CI)*	*4.0 (3.4–5.0)*	*PAF (%, 95% CI)*	*6.1 (5.6–7.5)*
		Q1	Reference	Q1	Reference	Q1	Reference
		Q2	1.03 (1.00–1.06)	Q2	1.07 (1.04–1.10)	Q2	1.06 (1.03–1.09)
		Q3	1.07 (1.04–1.09)	Q3	1.09 (1.06–1.12)	Q3	1.10 (1.08–1.13)
		Q4	1.14 (1.11–1.17)	Q4	1.21 (1.18–1.24)	Q4	1.19 (1.16–1.22)
		PAF (%, 95% CI)	5.7 (4.1–6.7)	PAF (%, 95% CI)	8.5 (6.9–9.5)	PAF (%, 95% CI)	8.2 (6.2–10.7)
4–14	35,719	*Normal*	*Reference*	*Normal*	*Reference*	*Normal*	*Reference*
		*Overweight*	*1.07 (1.04–1.09)*	*Increased*	*1.15 (1.13–1.18)*	*Increased*	*1.14 (1.11–1.16)*
		*Obese*	*1.18 (1.15–1.21)*	*NA*	*NA*	*NA*	*NA*
		*PAF (%, 95% CI)*	*6.9 (5.7–8.0)*	*PAF (%, 95% CI)*	*4.5 (4.0–5.1)*	*PAF (%, 95% CI)*	*6.7 (6.3–7.6)*
		Q1	Reference	Q1	Reference	Q1	Reference
		Q2	1.03 (1.00–1.06)	Q2	1.08 (1.05–1.11)	Q2	1.08 (1.05–1.12)
		Q3	1.08 (1.05–1.12)	Q3	1.12 (1.08–1.15)	Q3	1.13 (1.10–1.17)
		Q4	1.18 (1.14–1.21)	Q4	1.25 (1.21–1.29)	Q4	1.23 (1.19–1.27)
		PAF (%, 95% CI)	6.8 (5.4–8.6)	PAF (%, 95% CI)	10.2 (8.6–12.2)	PAF (%, 95% CI)	10.2 (8.3–12.1)
7–14	22,952	*Normal*	*Reference*	*Normal*	*Reference*	*Normal*	*Reference*
		*Overweight*	*1.06 (1.02–1.09)*	*Increased*	*1.16 (1.13–1.19)*	*Increased*	*1.15 (1.12–1.18)*
		*Obese*	*1.18 (1.14–1.23)*	*NA*	*NA*	*NA*	*NA*
		*PAF (%, 95% CI)*	*6.7 (3.2–9.4)*	*PAF (%, 95% CI)*	*4.7 (4.0–5.1)*	*PAF (%, 95% CI)*	*7.2 (5.6–8.2)*
		Q1	Reference	Q1	Reference	Q1	Reference
		Q2	1.02 (0.98–1.06)	Q2	1.09 (1.05–1.13)	Q2	1.09 (1.05–1.13)
		Q3	1.09 (1.04–1.13)	Q3	1.15 (1.10–1.19)	Q3	1.14 (1.10–1.19)
		Q4	1.19 (1.14–1.23)	Q4	1.27 (1.22–1.32)	Q4	1.25 (1.20–1.30)
		PAF (%, 95% CI)	7.1 (4.3–9.2)	PAF (%, 95% CI)	11.5 (9.0–13.6)	PAF (%, 95% CI)	10.8 (8.6–12.6)

The model was adjusted for age, sex, ethnicity, Townsend Deprivation Index, education, smoking status, alcohol intake, physical activity, dietary intake, menopause, hormone replacement therapy, regular use of nonsteroidal anti-inflammatory drugs, colorectal cancer screening, and mammography. Italicized text represents categories defined using standard cutoffs based on NHS criteria (Table S1), including BMI (normal: 18.5 to 24.9 kg/m^2^; overweight: 25.0 to 29.9 kg/m^2^; obesity: ≥30.0 kg/m^2^), waist circumference (men: >102 cm; women: >88 cm), and waist–hip ratio (men: >0.90; women: >0.85). CI, confidence interval; PAF, population attributable fraction; BMI, body mass index; NHS, National Health Survey

^a^ Cancer was defined according to the International Classification of Diseases, 10th Revision (ICD-10) codes C00 to C96, excluding C44 (non-melanoma skin cancer).

^b^ BMI quartiles are as follows: Q1 = less than 24.15 kg/m^2^, Q2 = 24.15 to less than 26.7 kg/m^2^, Q3 = 26.7 to less than 29.9 kg/m^2^, and Q4 = 29.9 kg/m^2^ or greater.

^c^ Waist circumference quartiles are as follows: Q1 = less than 89 cm for men and less than 75 cm for women, Q2 = 89 to less than 96 cm for men and 75 to less than 83 cm for women, Q3 = 96 to less than 103 cm for men and 83 to less than 92 cm for women, and Q4 = 103 cm or greater for men and 92 cm or greater for women.

^d^ Waist–hip ratio quartiles are as follows: Q1 = less than 0.89 for men and less than 0.77 for women, Q2 = 0.89 to less than 0.93 for men and 0.77 to less than 0.81 for women, Q3 = 0.93 to less than 0.98 for men and 0.81 to less than 0.86 for women, and Q4 = 0.98 or greater for men and 0.86 or greater for women.

Subgroup analyses showed patterns consistent with the main analyses across different follow-up periods. PAF estimates for adiposity measures appeared higher among women than men and among younger (<60 years) than older (≥60 years) participants (Fig. S2). Formal interaction analyses indicated evidence of interaction by sex for all adiposity measures except WC. Interaction by age was observed for WC and WHR, but not consistently for BMI (Table S5). Overall patterns were consistent, with central adiposity measures showing stronger associations than BMI.

Accounting for prediagnostic weight loss, risk below standard thresholds, and use of BMI as a suboptimal measure of adiposity increased PAF estimates, suggesting that the proportion of cancers attributable to excess weight may exceed previous estimates. In the UK, this proportion has been estimated at around 6.3% [2], whereas our estimates exceeded 10% when these factors were jointly considered. Similar patterns have previously been observed for colorectal cancer [7]. The current results suggest that these methodological factors may also be relevant to other cancers associated with excess weight. While this analysis focused on the overall cancer burden, future studies of site-specific PAFs may clarify which cancers drive it.

Prediagnostic weight loss during the preclinical phase can attenuate observed associations [9], with this phase potentially lasting up to 7 years, during which tumor-related processes may influence body weight [10]. To address this bias, we sequentially excluded the first 1 to 10 years of follow-up, assuming most baseline cancers would be diagnosed during this period. HR increased with longer exclusions, indicating progressive reduction of bias. Estimates for WC and WHR stabilized after approximately 7 years (Fig. S3), suggesting the bias had largely been removed. BMI-based estimates weakened, likely because BMI reflects both fat and lean mass and is more sensitive to cachexia-related changes. Previous cohort studies have inconsistently addressed reverse causation, typically excluding no more than the first 2 years of follow-up, which may be insufficient to address this bias. Excluding early follow-up may introduce selection bias due to noncancer mortality, but Fine and Gray competing risk models and applying lag periods yielded similar results, suggesting a limited impact.

Restricted cubic spline analyses (Fig. S4) indicate that cancer risk increases across adiposity measures, with steeper increases in women than in men. Except for BMI in men, risk increased monotonically from the 5th to the 95th percentile. These patterns, together with higher PAFs from quartile-based analyses, suggest that conventional thresholds may underestimate risk.

This study has several strengths. The large sample size, long follow-up, and use of multiple adiposity measures allowed for a comprehensive assessment of the cancer burden attributable to excess weight. Quartile-based analyses captured risk across the full distribution, and lag-time analyses reduced potential bias from prediagnostic weight loss. The distribution of obesity-related cancers in the UKB was similar to the general UK population (Table S6). Nonetheless, some limitations should be considered. PAF estimates depend on both the magnitude of the relative risk and the prevalence of the exposure. The UKB cohort is known to be healthier and less socioeconomically deprived than the general population, which may introduce selection bias and limit generalizability. The prevalence of adiposity (BMI and WC) is higher in the general UK population than in the UKB cohort. Applying the relative risk estimates derived from our analysis to these higher adiposity prevalences, therefore, leads to larger PAF estimates (Table S7). However, PAFs assume a causal relationship between adiposity and cancer. As our analyses are based on observational data, HRs may not fully reflect causal effects due to potential residual confounding, measurement error, and model assumptions. Adiposity was assessed only at baseline, and changes in body weight or fat distribution over time were not captured, which may have led to exposure misclassification and attenuation of associations. Future analyses incorporating repeated measurements may provide more accurate estimates of long-term risk.

These findings highlight the urgent need to increase efforts to cope with and overcome the obesity epidemic, which is ongoing in most countries around the globe, to alleviate the expected major rise in the cancer burden that will result from the demographic changes in the years and decades to come.

## Ethical Approval

This research was conducted using the UK Biobank Resource under application no. 66591. The UK Biobank has received ethical approval from the North West Multi-Centre Research Ethics Committee. All participants provided written informed consent prior to enrollment.

## Data Availability

Data were reused with the permission of the UK Biobank. The UK Biobank is an open-access resource, and bona fide researchers can apply to use the UK Biobank dataset by registering and applying at https://www.ukbiobank.ac.uk/use-our-data/research-analysis-platform/. The data and analysis codes used for this study will be available on the UK Biobank website for registered UK Biobank researchers, subject to an application fee.
