# Supplementary material for: Excess Weight May Account for More Than 10% of All Cancers: The Underestimated Impact of the Obesity Epidemic
Source: Cancer Commun (Lond). 2026 Jul 2;46:0040. doi: 10.34133/cancomm.0040 (PMC13324164; doi:10.34133/cancomm.0040)
Supplement: Supplementary 1 — Figs. S1 to S4 Tables S1 to S7 [file cancomm.0040.f1.pdf]

Supplementary Materials for

Excess Weight May Account for More than Ten Percent of all Cancers: The Underestimated Impact of the Obesity Epidemic

Luna Kiran Adhikari[1,2,3], Fatemeh Safizadeh[4], Marko Mandic[4], Hermann Brenner[4,*]

[1]Division of Clinical Epidemiology of Early Cancer Detection, German Cancer Research Centre (DKFZ), Heidelberg, Germany

[2]Institute for Medical Information Processing, Biometry and Epidemiology (IBE), Faculty of Medicine, LMU Munich, Munich, Germany

[3]Pettenkofer School of Public Health, Munich, Germany

[4]Cancer Prevention Graduate School, German Cancer Research Centre (DKFZ), Heidelberg, Germany

Corresponding author:

Hermann Brenner, Cancer Prevention Graduate School, German Cancer Research Centre (DKFZ), Heidelberg, Germany; h.brenner@dkfz.de

Rationale:

A large body of epidemiologic evidence has reported consistent associations between body mass index and site specific cancers such as colorectal [1], breast [2] , endometrial [3], and pancreatic cancer [4], among others [5,6] as well as evidence supporting the role of central adiposity measures [7–9], including waist circumference and waist-to-hip ratio, in cancer risk. Although these relationships are well documented, relatively few studies have quantified the proportion of overall cancer cases attributable to excess weight. Available estimates of population attributable fractions (PAFs) suggest that approximately 2% to 8% of all cancer cases are attributable to excess body weight, with variation by sex, region, and cancer site [10–13]. However, most of these estimates rely on body mass index (BMI) as the sole proxy for adiposity, typically using continuous measures or standard BMI categories, and often do not account for prediagnostic weight loss, which may attenuate observed associations.

The present analysis builds on this literature by quantifying how central obesity adiposity measures including waist circumference (WC) and waist-hip ratio (WHR), methodological factors including prediagnostic weight loss and exposure categorization, may influence estimates of the overall cancer burden associated with excess adiposity.

Methodology:

Cancer Incidence- Incident cancer cases were identified through linkage with national cancer registries in England, Wales, and Scotland within the UK Biobank database. Cancer diagnoses were defined using the International Classification of Diseases, 10th Revision (ICD-10). Overall cancer was defined as ICD-10 codes C00–C96, excluding C44 (non-melanoma skin cancer). These cancer registries gather information on cancer diagnoses from multiple sources (hospitals, cancer centres and treatment centres, hospices and nursing homes, private hospitals, cancer screening programmes, other cancer registers, general practices, death certificates, Hospital Episode Statistics (HES), and Cancer Waiting Time (CWT). The UK Biobank (UKB) received ethical approval from the North West Multi-Centre Research Ethics Committee, and all participants provided written informed consent.

Participants- Participants were excluded if they had a history of cancer at baseline (according to national cancer registry records) before or at the initial assessment visit. Individuals were also excluded if data on BMI, WC, or WHR were missing. Participants were followed prospectively from baseline until cancer diagnosis, death, loss to follow-up, or the end of registry follow-up, whichever occurred first. Follow-up was complete through February 29, 2020, in England; December 31, 2016, in Wales; and January 31, 2021, in Scotland.

Procedures- At baseline, participants completed self-administered questionnaires on sociodemographic characteristics, lifestyle behaviours, and medical history, followed by nurse-led interviews and physical measurements. Trained staff measured height,

weight, waist, and hip circumferences using standardised protocols and calibrated equipment.

- BMI: Calculated as weight in kilograms divided by height in meters squared (kg/m^2). BMI was classified into quartiles based on the study population and according to National Health Survey (NHS) England reporting standards [14]: normal (18.5 - 24.9 kg/m^2), overweight (25.0 - 29.9 kg/m^2), and obese ($\geqslant$30.0 kg/m^2). Participants who were underweight (<18·5 kg/m^2) were grouped with the normal category due to very small numbers (n = 2,353, 0.51 % of the cohort).
- WC was categorized into sex-specific quartiles. Alternatively, following the 2010 NHS England reporting standards [14], a raised WC was defined as >102 cm for men and >88 cm for women.
- WHR was calculated as waist divided by hip circumference (cm) and categorized into sex-specific quartiles and 2010 NHS England reporting standards: normal ($\leqslant$0.90 men, $\leqslant$0.85 women) and high (>0.90 men, >0.85 women).

Quartile-based categorization was used to capture the full distribution of adiposity within the cohort and to assess risk gradients beyond conventional clinical thresholds, which may underestimate risk associated with increasing adiposity. Using quartiles also allowed the three adiposity measures to be evaluated on a comparable scale despite their different units and clinical cut-offs. This approach was intended to examine exposure-response relationships across the distribution. Although measurements were conducted by trained staff, some measurement variability may still be present which may have influenced the estimated associations.

Statistical Analyses:

Analyses were performed using SAS 9.4 (SAS Institute Inc) and R 4.3.2 (R Foundation). Baseline characteristics of the overall cohort and participants with incident cancer were summarized as medians with interquartile ranges (IQR) for continuous variables and as frequencies with percentages for categorical variables. Missing covariate data were handled using multiple imputation by chained equations (mice package in R). Five imputed datasets were generated, and results were combined using Rubin’s rules.

Cox proportional hazards models estimated hazard ratios (HRs) and 95% confidence intervals (CIs) for the associations between adiposity measures (BMI, WC, and WHR) and overall cancer risk. The lowest quartile was used as the reference for all exposures. The proportional hazards assumption was evaluated using Schoenfeld residuals, and no violations were detected. To investigate potential non-linear dose–response relationships, we fitted sex-specific restricted cubic spline models. Knots were positioned at the 5th, 25th, 50th, 75th, and 95th percentiles of each adiposity measure, and the 25th percentile was used as the reference. Subgroup analyses were

conducted by age (<60 vs ⩾60 years) and sex. Potential modification by the stratification variables was evaluated by including an interaction term of each anthropometric measure (continuous) and the stratification variables in the model.

Analyses Excluding Early Follow-up:

To address potential bias from prediagnostic weight loss, analyses were first conducted using the full follow-up period and then repeated after sequentially excluding the first 1 to 10 years of follow-up. Weight loss may occur during the preclinical phase of cancer, when tumors are present but not yet clinically diagnosed [15]. Evidence suggests that the sojourn time for many cancers can extend up to approximately seven years, during which tumor-related metabolic and inflammatory processes may already influence body weight [16]. Such changes can shift individuals from higher to lower BMI or central adiposity categories before diagnosis, weakening the observed association between excess weight and cancer risk. Since our outcome included all types of cancers, excluding the first seven years of follow-up therefore represents a conservative approach to minimize bias from disease-related weight loss. To further evaluate the stability of the findings, we also assessed a four-year lag period, representing a shorter exclusion window. Examining both shorter and longer lag periods allows assessment of whether the observed associations are robust to different assumptions regarding the potential duration of preclinical disease.

Population Attributable Fraction:

PAFs and 95% confidence intervals were estimated using the graphPAF R package with 1,000 bootstrap replications [17]. The lowest category of the adiposity measures was used as the reference. The total PAF represents the proportion of cases that could be prevented if all individuals were in the lowest category. PAFs were calculated using a Levin-type formula based on adjusted hazard ratios:

$$PAF=\frac{\sum_i P_i\,(RR_i-1)}{1+\sum_i P_i\,(RR_i-1)}$$

where:

- P_i represents the prevalence of exposure category *i*
- RR_i represents the adjusted hazard ratio for that category relative to the reference group.

PAFs were calculated for:

- the full follow-up period (0–14 years)
- analyses excluding early follow-up years (4 years and 7 years)

- standard adiposity categories using population prevalence estimates from NHS England [18]. To ensure comparability with the UKB, prevalence values were calculated as the average across 2006-2010, corresponding to the Biobank recruitment period. For these analyses, population prevalence estimates from NHS England (2010) were combined with adjusted hazard ratios derived from the UKB cohort to formally recalculate population attributable fractions using a Levin-type formula.

Interpretation of PAF estimates relies on the assumption of a causal relationship between exposure and outcome, that confounding has been adequately controlled, and that the statistical model is correctly specified. However, as this study is based on observational data, these assumptions cannot be fully verified, and residual confounding or bias may influence the estimated hazard ratios and corresponding PAFs.

Covariates:

All covariates were derived from baseline assessments in the UK Biobank.

Educational attainment was derived from UK Biobank Data Fields 6138 and 10722 and categorized as: higher academic qualifications (college/university degree, A-levels/AS-levels, or other higher qualifications), lower academic/vocational qualifications (O-levels/GCSEs, CSEs, or vocational qualifications), or none (including negative responses).

Deprivation Index: Socioeconomic deprivation was measured using the Townsend Deprivation Index at recruitment. This continuous variable reflects area-level deprivation derived from national census data based on the participant’s residential postcode.

Physical activity was assessed using adapted questions from the validated short-form International Physical Activity Questionnaire (IPAQ), which captures the frequency and duration of walking, moderate, and vigorous activities. Participants were categorized as low, moderate, or high physical activity according to standard IPAQ scoring criteria based on total weekly metabolic equivalent (MET)-minutes and activity frequency.

Nonsteroidal anti-inflammatory drug (NSAID) or aspirin use was defined using both self-reported medication data and linked prescription records. Participants were classified as users (yes) if regular use of aspirin or other NSAIDs was reported in either the questionnaire data or prescription records. Participants were classified as non-users (no) only when both data sources indicated no NSAID or aspirin use.

A diet score was constructed using self-reported frequency of intake for: Fruit (fresh and dried), vegetables (raw, cooked), processed meat, unprocessed red meat (beef, lamb, pork), fish (oily and non-oily), whole grains (e.g., bran, oats, wholemeal bread), and refined grains (e.g., cereal, white/brown bread). A point was given for each healthy dietary criterion met: Fruit $\geqslant$3 servings/day, Vegetables $\geqslant$3 servings/day, Processed

meat ⩽1 serving/week, Unprocessed red meat ⩽1.5 servings/week, Fish ⩾2 servings/week, Whole grains ⩾3 servings/day, Refined grains ⩽1.5 servings/day. The total score (0–7) was categorized as: Low (0–1 points), Intermediate (2–5 points), High (6–7 points)

Although we adjusted for a broad range of potential confounders, some relevant factors, such as detailed dietary patterns, inflammatory markers, comorbidities, and weight history, were not fully captured or incorporated into the analysis. These factors may influence both adiposity and cancer risk and could contribute to residual confounding.

Supplementary Tables

Supplementary Table S1. Categorization of BMI, WC and WHR according to National Health Survey England reporting standard

Measure	Category	Men	Women
**BMI (kg/m²)**	Normal	18.5–24.9	18.5–24.9
	Overweight	25.0–29.9	25.0–29.9
	Obese	⩾30.0	⩾30.0
**Waist circumference (cm)**	Raised WC	>102	>88
**Waist–hip ratio**	Normal	⩽0.90	⩽0.85
	High	>0.90	>0.85

BMI, WC, and WHR categories were defined according to National Health Survey England reporting standards. Source: Available at: https://data.parliament.uk/DepositedPapers/Files/DEP2012-1861/132327-10.pdf. Last accessed 11 June 2026.

Abbreviations: BMI, body mass index; WC, waist circumference; WHR, waist-hip ratio.

Supplementary Table S2. Subdistribution hazard ratios (95% CI) for cancer incidence by adiposity measures across follow-up periods, accounting for the competing risk of death

**Follow-up period, years**	**Number of Cancer[a] cases**	**HR (95% CI)**			
		**Quartile[b]**	**BMI**	**WC**	**WHR**
0-14	50,136	Q1	Reference	Reference	Reference
		Q2	1.03 (1.00-1.06)	1.07 (1.04-1.10)	1.06 (1.03-1.09)
		Q3	1.07 (1.04-1.10)	1.09 (1.06-1.12)	1.10 (1.07-1.13)
		Q4	1.13 (1.11-1.17)	1.20 (1.17-1.23)	1.18 (1.14-1.21)
4-14	35,719	Q1	Reference	Reference	Reference
		Q2	1.03 (1.00-1.06)	1.08 (1.05-1.11)	1.08 (1.05-1.12)
		Q3	1.08 (1.05-1.12)	1.12 (1.08-1.15)	1.13 (1.09-1.17)
		Q4	1.17 (1.13-1.21)	1.24 (1.20-1.27)	1.23 (1.18-1.25)
7-14	22,952	Q1	Reference	Reference	Reference
		Q2	1.02 (1.00-1.05)	1.09 (1.05-1.13)	1.09 (1.05-1.12)
		Q3	1.09 (1.04-1.13)	1.15 (1.10-1.19)	1.14 (1.09-1.18)
		Q4	1.18 (1.13-1.23)	1.26 (1.21-1.31)	1.24 (1.19-1.28)

The model was adjusted for age, sex, ethnicity, Townsend deprivation index, education, smoking status, alcohol intake, physical activity, dietary intake, menopause, hormonal replacement therapy, regular use of nonsteroidal anti-inflammatory drugs, colorectal cancer screening, and mammography.

Estimates were derived using a Fine and Gray model, with death from any cause as the competing event.

[a]Cancer defined as: C00–C96, excluding C44 (non-melanoma skin cancer).

[b]Quartiles are as follows:

BMI: Q1 = less than 24.15 kg/m^2, Q2 = 24.15 to less than 26.7 kg/m^2, Q3 =26.7 to less than 29.9 kg/m^2, Q4 = 29.9 kg/m^2 or greater;

Waist circumference: Q1 = less than 89 cm for men and less than 75 cm for women, Q2 = 89 to less than 96 cm for men and 75 to less than 83 cm for women, Q3 = 96 to less than 103 cm for men and 83 to less than 92 cm for women, Q4 = 103 cm or greater for men and 92 cm or greater for women;

Waist-hip ratio: Q1 = less than 0.89 for men and less than 0.77 for women, Q2 = 0.89 to less than 0.93 for men and 0.77 to less than 0.81 for women, Q3 = 0.93 to less than 0.98 for men and 0.81 to less than 0.86 for women, Q4 = 0.98 or greater for men and 0.86 or greater for women.

Abbreviations: CI, confidence interval; BMI, body mass index; WC, waist circumference; WHR, waist-hip ratio; HR, hazard ratio

Supplementary Table S3. Baseline characteristics of the study population

**Characteristics**	**Categories**	**Total cohort (*n* = 458,543)**	**Incident cancer cases (*n* = 50,136)**
Age at baseline, years	NA	57 (50, 63)	61 (56, 65)
BMI	NA	26.7 (24.2, 29.9)	26.7 (24.1, 29.9)
Waist circumference	Women	83.0 (75.0, 92.0)	85.0 (77.0, 94.0)
	Men	96.0 (89.0, 103.0)	97.0 (90.0, 105.0)
Waist-hip ratio	Women	0.81 (0.76, 0.86)	0.82 (0.77, 0.87)
	Men	0.93 (0.89, 0.97)	0.94 (0.90, 0.98)
Sex	Women	244,351 (53.3%)	23,159 (46.2%)
	Men	214,192 (46.7%)	26,977 (53.8%)
Ethnicity	White	431,110 (94.0%)	48,110 (95.9%)
	Mixed or other	6,988 (1.5%)	527 (1.1%)
	Asian or Chinese	10,747 (2.4%)	670 (1.3%)
	Black	7,494 (1.6%)	585 (1.2%)
Townsend Deprivation Index	NA	-2.14 (-3.65, 0.54)	-2.19 (-3.67, 0.54)
Education Category	Higher academic	224,480 (48.9%)	22,777 (45.4%)
	Lower academic	152,042 (33.2%)	15,745 (31.4%)
	None	76,593 (16.7%)	10,981 (21.9%)
Smoking Status	Never smoker	251,721 (54.9%)	23,646 (47.2%)
	Previous smoker	156,202 (34.1%)	19,667 (39.2%)
	Current smoker	48,339 (10.5%)	6,521 (13.0%)
Alcohol Intake	Daily	93,176 (20.3%)	11,646 (23.2%)
	Three or four times a week	106,422 (23.2%)	11,450 (22.8%)
	Once or twice a week	118,367 (25.8%)	12,298 (24.5%)
	One to three times a month	50,981 (11.1%)	5,053 (10.1%)
	Special Occasion only	52,117 (11.4%)	5,684 (11.3%)
	Never	36,458 (8.0%)	3,894 (7.8%)
	Low	64,909 (14.2%)	7,303 (14.6%)

Physical Activity	Moderate	143,279 (31.3%)	15,623 (31.2%)
	High	144,780 (31.6%)	15,193 (30.3%)
Dietary Intake	Wholegrains ⩾3 servings/day	50,334 (11.0%)	5,838 (11.6%)
	Refined grains ⩽ 1.5 servings/day	186,219 (40.6%)	19,792 (39.5%)
	Red meat ⩽1.5 servings/week	299,003 (65.2%)	31,151 (62.1%)
	Processed meat ⩽ 1 serving/week	313,866 (68.5%)	33,557 (66.9%)
	Vegetable ⩾3 servings/day	372,060 (81.2%)	40,690 (81.2%)
	Fruit ⩾3 servings/day	227,995 (49.7%)	24,384 (48.6%)
Menopause (women only)	Yes	145,695 (31.8%)	15,821 (31.6%)
HRT (women only)	Yes	91,570 (20.0%)	10,306 (20.6%)
Regular use of NSAIDs or aspirin	Yes	140,620 (30.7%)	16,700 (33.3%)
Colorectal cancer screening	Yes	137,093 (29.9%)	18,416 (36.7%)
Mammography	Yes	191,721 (41.8%)	25,007 (49.9%)

Data are expressed as median (Q1, Q3) or number of participants (percentage). Percentages might not add up to 100 percent due to rounding.

Numbers of missing values in the total cohort are as follows: age: 0, sex: 0, ethnicity: 2,204, Townsend Deprivation Index: 564, education category: 5,428, smoking status: 2,281, alcohol intake: 1,022, physical activity: 105,575, dietary intake: 614, menopausal status: 752, HRT use:1,263, regular use of NSAIDs or aspirin: 16, colorectal cancer screening: 8,494, mammography:749. Abbreviations: HRT, hormone replacement therapy; NSAIDS, nonsteroidal anti-inflammatory drugs; NA, not applicable.

Supplementary Table S4. Impact of categorization and follow-up exclusion on population attributable fractions

Adiposity measure	PAF (%) - standard categorization[a], 0 to14 year follow-up	PAF (%) - quartile categorization, 0 to 14 year follow-up	PAF (%) quartile - categorization, 7 to 14 year follow-up	Absolute difference[b]
BMI	5.5	5.7	7.1	+ 1.6% units
Waist-hip Ratio	6.1	8.2	10.8	+ 5.3% units
Waist circumference	4.0	8.5	11.5	+ 6.0% units

[a] Standard categorization was based on established cutoffs:

- BMI: Normal (18.5–24.9 kg/m²), Overweight (25.0–29.9 kg/m²), Obese (⩾30.0 kg/m²); underweight (<18.5 kg/m²) were grouped with the normal category due to very small numbers.
- Waist circumference: High (>102 cm in men, >88 cm in women), Normal (⩽102 cm in men, ⩽ 88 cm in women)
- Waist-hip ratio: High (>0.90 in men, >0.85 in women), Normal (⩽0.90 in men, ⩽0.85 in women).

[b]Absolute difference was calculated relative to the reference analysis (BMI using standard categorization and the full 0 to 14 year follow-up period; PAF = 5.5%), which represents the most

commonly used approach in the literature. Values therefore represent the difference between the PAF obtained using quartile categorization with restriction to follow-up years 7 to 14 and the reference PAF.

Abbreviations: PAF, population attributable fraction; BMI, body mass index.

Supplementary Table S5. *P* values for interaction between adiposity measures, sex and age in relation to cancer risk

Followup period, years	Exposure	Interaction with sex	Interaction with age
0 to 14	BMI	<0.001	0.858
	WC	0.024	<0.001
	WHR	<0.001	<0.001
4 to 14	BMI	<0.001	0.468
	WC	0.033	<0.001
	WHR	<0.001	<0.001
7 to 14	BMI	<0.001	0.750
	WC	0.165	0.002
	WHR	<0.001	<0.001

P values for interaction were obtained by including cross-product terms between each adiposity measure and sex or age group in multivariable Cox proportional hazards models. Models were adjusted for age, sex, ethnicity, Townsend deprivation index, education, smoking status, alcohol intake, physical activity, dietary intake, menopausal status, hormone replacement therapy use, regular use of nonsteroidal anti-inflammatory drugs, colorectal cancer screening, and mammography. Age was categorized as <60 and ⩾60 years. Follow-up periods reflect exclusion of the first 0, 4, and 7 years to account for potential bias due to prediagnostic weight loss. Abbreviations: BMI, body mass index; WC, waist circumference; WHR, waist-hip ratio.

Supplementary Table S6: Obesity related cancer site distribution in the study population compared with the UK general population [19]

Site	ICD-10 code	Cancer events, *n* (UKB)	Incidence share (%), UKB	Incidence share %, UK general population (2018-2019, 2021)
Breast	C50.0-C50.6, C50.8, C50.9	3,240	14.1	15.0
Colorectal	C18.0- C18.9, C19, C20	2,612	11.4	12.0
Endometrial	C54.1	624	2.7	3.0
Kidney	C64	693	3.0	4.0
Pancreatic	C25.0-C25.4, C25.7, C25.8, C25.9	664	2.9	3.0
Oesophageal	C15.0-C15.5, C15.8, C15.9	512	2.2	2.0
Ovarian	C56	443	1.9	4.0
Multiple myeloma	C90.0	447	1.9	2.0
Liver	C22.0, C22.1, C22.3, C22.4, C22.7, C22.9	340	1.5	2.0
Thyroid	C73	197	0.9	1.0
Stomach	C16.0	137	0.6	2.0
Gallbladder	C23	56	0.2	<1.0
Meningioma	C70.0, C70.9	4	0.1	ND
Others	NA	12961	56.5	ND

Incidence share (%) was calculated based on cancer cases occurring after exclusion of the first 7 years of follow-up to reduce potential bias from reverse causation (prediagnostic weight loss). UK-specific incidence share represents the proportion of newly diagnosed cancer cases by site in the general UK

population. The incidence share for stomach cancer in the UK general population is based on all stomach cancer subsites (ICD-10 C16), whereas the UKB analysis includes only cardia stomach cancer (C16.0); this difference in case definition may result in non-comparable proportions between the two populations.

Abbreviations: UKB, UK Biobank; ICD-10, International Classification of Diseases, 10th Revision; ND, not defined; NA, not applicable.

Supplementary Table S7. Differences in the prevalence of adiposity in the UKB dataset and NHS England [18] and their implications for PAF estimates

	**UKB**			**NHS**		
	**Men (%)**	**Women (%)**	**PAF[a] % (95%CI)**	**Men (%)**	**Women (%)**	**PAF[a] % (95% CI)**
**BMI[b]**						
Normal	24.9	39.7	5.5 (3.9-6.9)	22.1	34.2	6.5 (3.6-9.4)
Overweight	49.3	36.6		47.3	35.5	
Obese	25.4	23.5		30.1	29.2	
**Waist circumference[c]**						
Raised Waist	27.4	33.4	4.0 (3.4-5.0)	42.9	51.5	6.4 (4.6-8.2)

Waist-hip ratio prevalence was not reported in the NHS, so prevalence estimates are assumed to be underestimated, similarly to waist data in UKB.

[a]Prevalence estimates shown for UKB were calculated among participants included in the analytical cohort, comprising 214,192 men and 244,351 women. NHS prevalence estimates were obtained from the National Health Survey for England and averaged across survey years 2006–2010 to correspond to the UKB recruitment period. PAFs were recalculated by combining population prevalence estimates with adjusted hazard ratios from the UKB cohort using the Levin formula and was calculated for full follow-up years.

[b]Normal weight if BMI was from 18.5 to less than 25kg/m^2; overweight if BMI was from 25 to less than 30kg/m^2; obese, if BMI was more than 30 kg/m^2; underweight (<18.5 kg/m^2) were grouped with the normal category due to very small numbers.

[c]Raised waist circumference if greater than 102 cm for men and greater than 88 cm for women.

Abbreviations: CI, confidence interval; PAF, population attributable fraction; UKB, UK biobank; NHS, national health survey.

Supplementary Figures

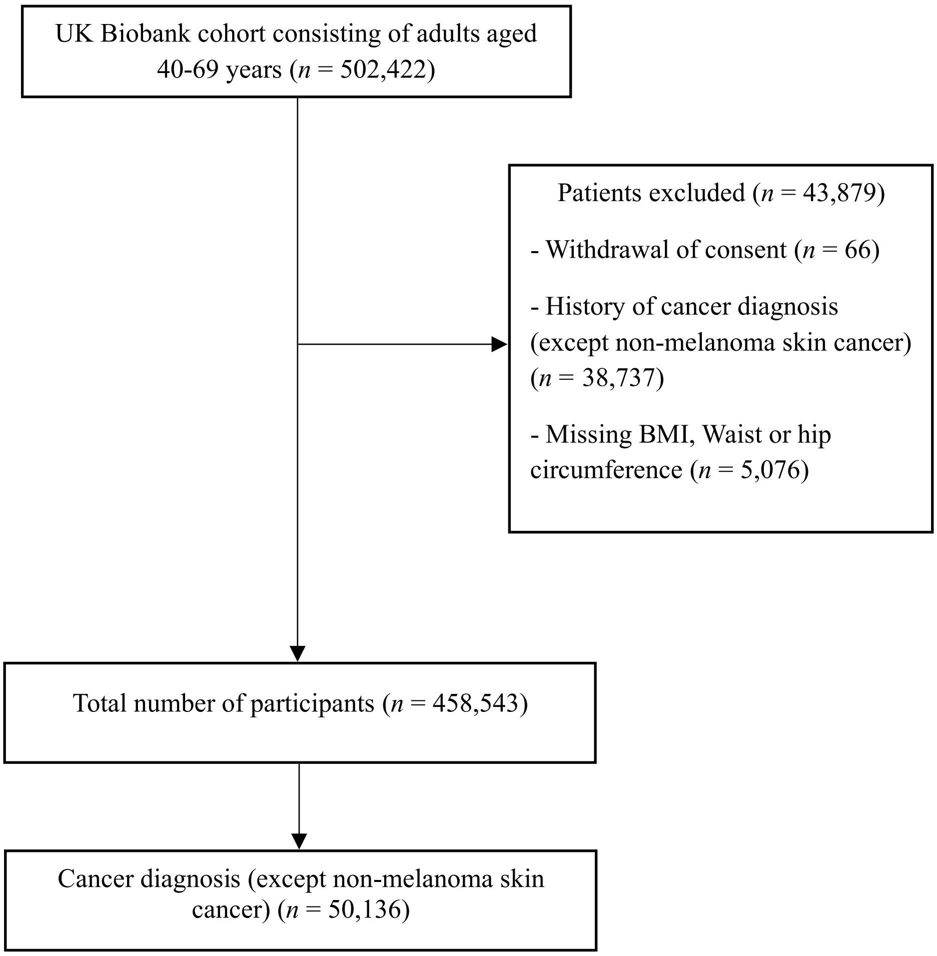

Supplementary Figure S1. Flowchart of the selection of the study population

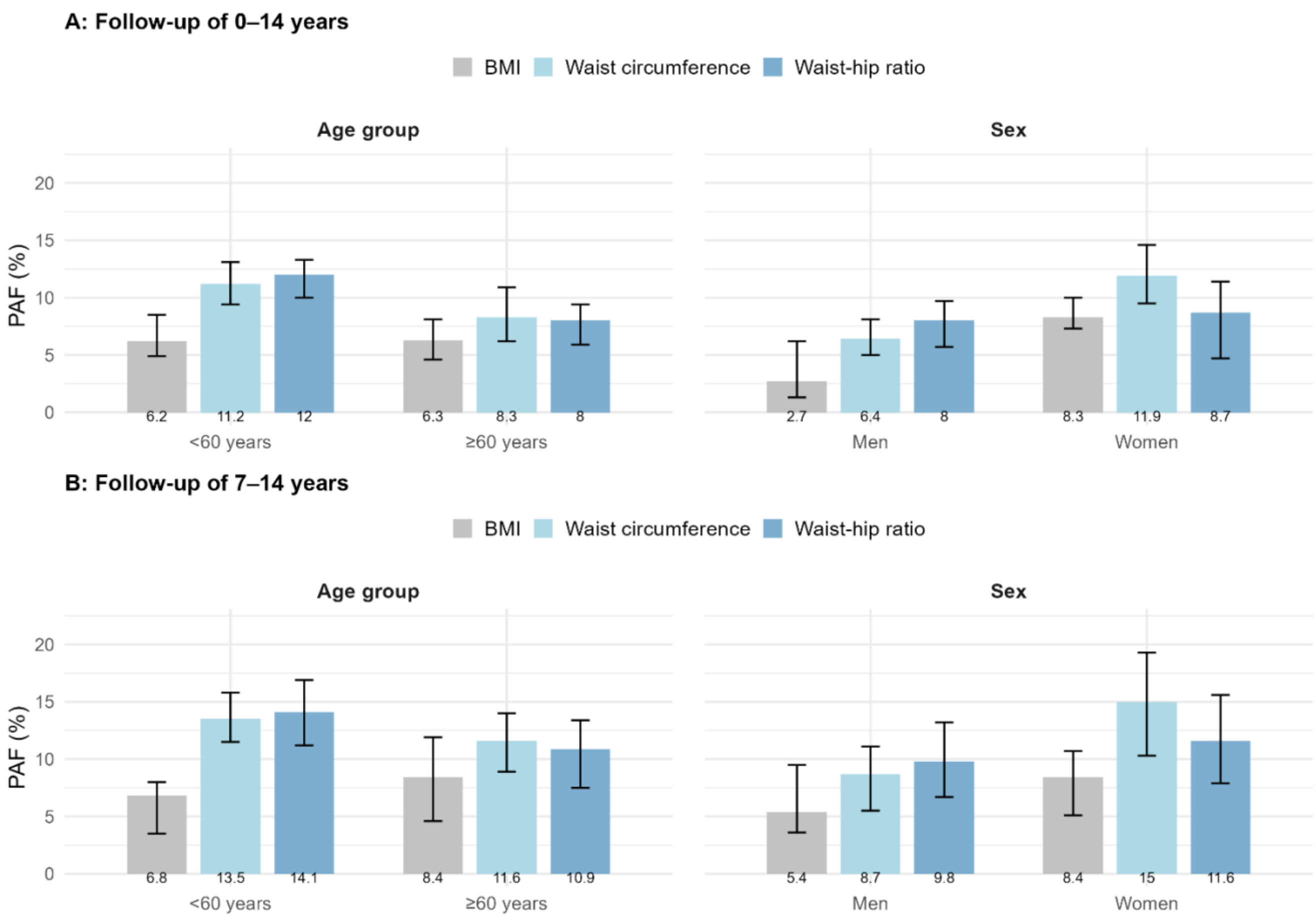

Supplementary Figure S2. PAFs for cancer incidence by adiposity measures across age, sex, and follow-up.

Error bars indicate 95% CIs. Abbreviations: BMI, body mass index; PAF, population attributable fraction.

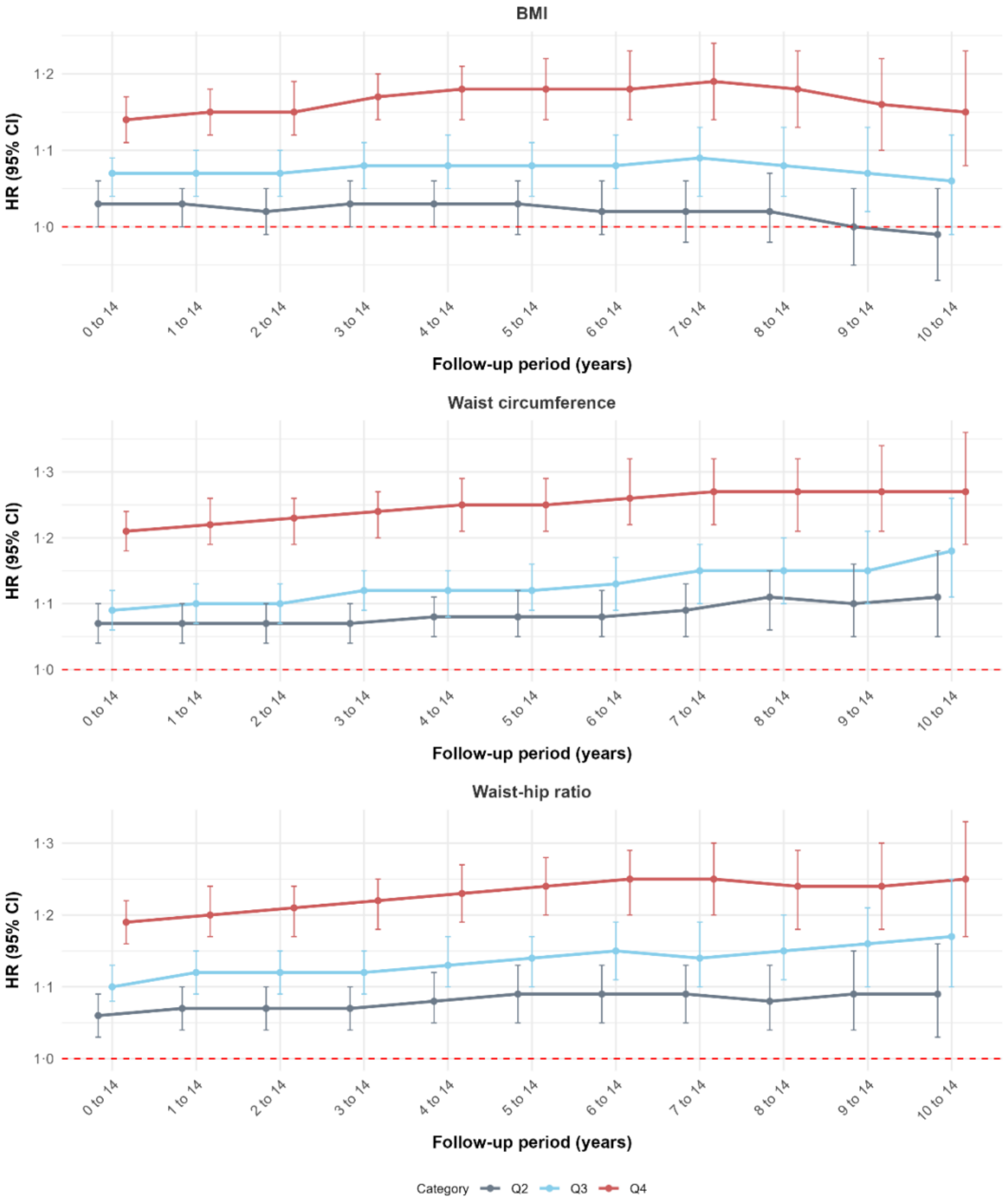

Supplementary Figure S3. Association Between Adiposity Measures and Cancer Risk Across Follow-Up Periods.

Model adjusted for age, sex, ethnicity, Townsend deprivation index, education, smoking status, alcohol intake, physical activity, dietary intake, menopause, hormonal replacement therapy, regular use of nonsteroidal anti-inflammatory drugs, colorectal cancer screening, and mammography.

BMI quartiles are as follows, Q1= less than 24.15 kg/m^2, Q2 = 24.15 to less than 26.7 kg/m^2, Q3 =26.7 to less than 29.9 kg/m^2, Q4 = 29.9 kg/m^2 or greater.

Waist circumference quartiles are as follows: Q1 = less than 89 cm for men and less than 75 cm for women, Q2 = 89 to less than 96 cm for men and 75 to less than 83 cm for women, Q3 = 96 to less than 103 cm for men and 83 to less than 92 cm for women. Q4 = 103 cm or greater for men and 92 cm or greater for women.

Waist-hip ratio quartiles are as follows: Q1 = less than 0.89 for men and less than 0.77 for women, Q2 = 0.89 to less than 0.93 for men and 0.77 to less than 0.81 for women, Q3 = 0.93 to less than 0.98 for men and 0.81 to less than 0.86 for women, Q4 = 0.98 or greater for men and 0.86 or greater for women.

Error bars indicate 95% CIs.

Abbreviations: BMI, body mass index; CI, confidence interval; HR, Hazard Ratio

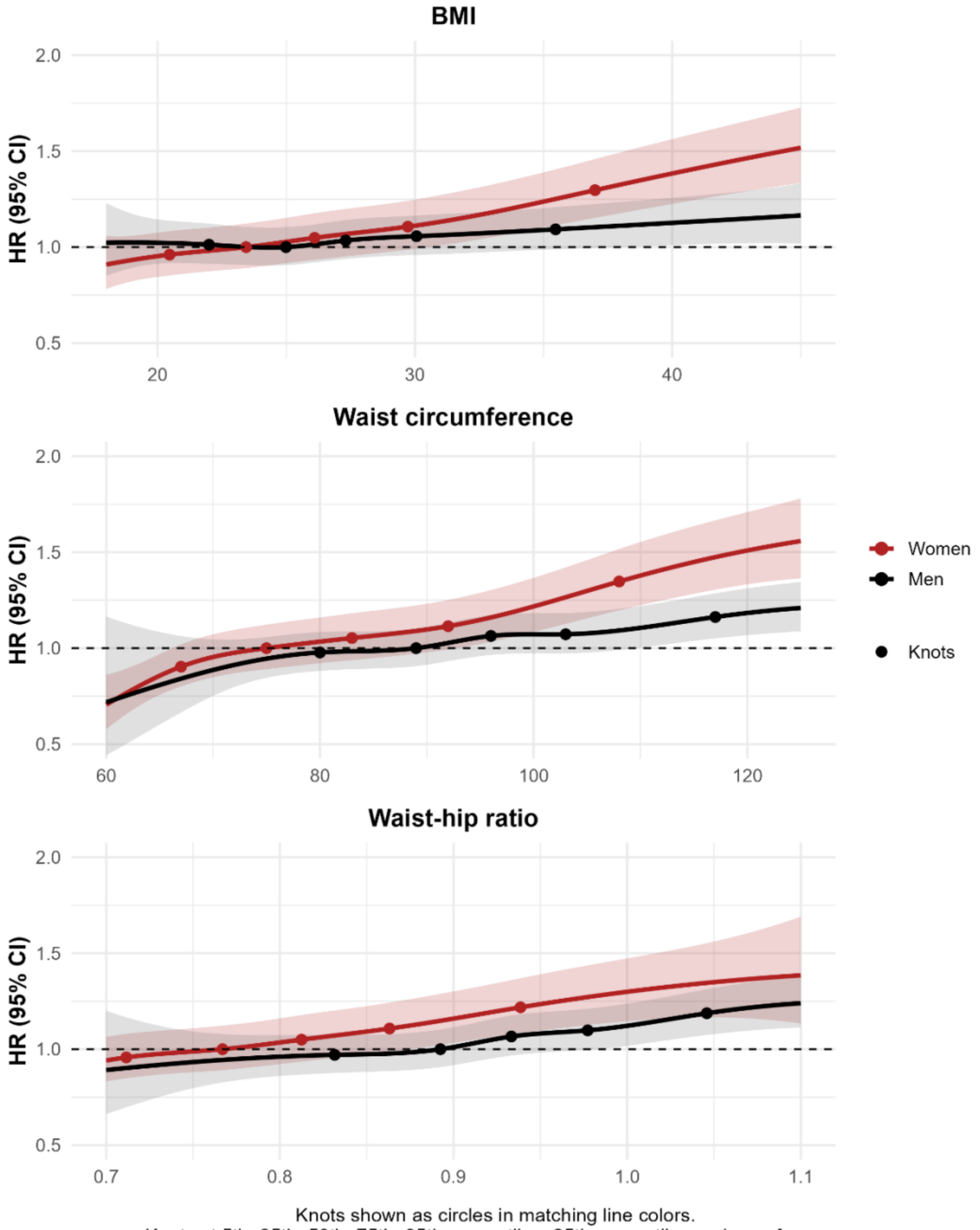

Supplementary Figure S4. Restricted cubic spline analysis stratified by sex

Model adjusted for age, sex, ethnicity, Townsend deprivation index, education, smoking status, alcohol intake, physical activity, dietary intake, regular use of nonsteroidal anti-inflammatory drugs, colorectal cancer screening for men.
Model adjusted for age, sex, ethnicity, Townsend deprivation index, education, smoking status, alcohol intake, physical activity, dietary intake, menopause, hormonal replacement therapy, regular use of nonsteroidal anti-inflammatory drugs, colorectal cancer screening, mammography for women.

Abbreviations: BMI, body mass index; HR, Hazard ratio; CI, Confidence interval.

References

[1] Leung LJC-L, Sharma RS, Cheng B, Akalanka HK, Gopalan V. Obesity and colorectal cancer risk: A systematic review and meta-analysis. World J Clin Oncol 2026;17:112369. doi: 10.5306/wjco.v17.i1.112369.

[2] Dehesh T, Fadaghi S, Seyedi M, Abolhadi E, Ilaghi M, Shams P, et al. The relation between obesity and breast cancer risk in women by considering menstruation status and geographical variations: a systematic review and meta-analysis. BMC Womens Health 2023;23:392. doi: 10.1186/s12905-023-02543-5.

[3] Onstad MA, Schmandt RE, Lu KH. Addressing the role of obesity in endometrial cancer risk, prevention, and treatment. J Clin Oncol 2016;34:4225–30. doi: 10.1200/JCO.2016.69.4638.

[4] Klein AP. Pancreatic cancer epidemiology: understanding the role of lifestyle and inherited risk factors. Nat Rev Gastroenterol Hepatol 2021;18:493. doi: 10.1038/s41575-021-00457-x.

[5] Pati S, Irfan W, Jameel A, Ahmed S, Shahid RK. Obesity and Cancer: A Current Overview of Epidemiology, Pathogenesis, Outcomes, and Management. Cancers 2023, Vol 15, 2023;15. doi: org/10.3390/cancers15020485.

[6] Sun M, da Silva M, Bjørge T, Fritz J, Mboya IB, Jerkeman M, et al. Body mass index and risk of over 100 cancer forms and subtypes in 4.1 million individuals in Sweden: the Obesity and Disease Development Sweden (ODDS) pooled cohort study. Lancet Reg Heal - Eur 2024;45:101034. doi: 10.1016/j.lanepe.2024.101034.

[7] Motuma A, Mussa I, Deressa A, Regassa LD, Birhanu A. Central obesity increases the risk of breast cancer irrespective of menopausal status in women: Systematic review and meta-analysis. Cancer Treat Res Commun 2025;44:100965. doi: 10.1016/j.ctarc.2025.100965.

[8] Du X, Hidayat K, Shi BM. Abdominal obesity and gastroesophageal cancer risk: systematic review and meta-analysis of prospective studies. Biosci Rep 2017;37:BSR20160474. doi: 10.1042/BSR20160474.

[9] Sun M, Häggström C, Da Silva M, Mboya IB, Lagerros YT, Michaëlsson K, et al. Comparing waist circumference with body mass index on obesity-related cancer risk: a pooled Swedish study. JNCI J Natl Cancer Inst 2025;117:1999–2009. doi: 10.1093/jnci/djaf075.

[10] Brown KF, Rumgay H, Dunlop C, Ryan M, Quartly F, Cox A, et al. The fraction of cancer attributable to modifiable risk factors in England, Wales, Scotland, Northern Ireland, and the United Kingdom in 2015. Br J Cancer 2018;118:1130. doi: 10.1038/s41416-018-0029-6.

[11] Fink H, Langselius O, Vignat J, Rumgay H, Rehm J, Martinez RX, et al. Global and regional cancer burden attributable to modifiable risk factors to inform prevention. Nat Med 2026 2026:1–10. doi: 10.1038/s41591-026-04219-7.

[12] Islami F, Goding Sauer A, Miller KD, Siegel RL, Fedewa SA, Jacobs EJ, et al. Proportion and number of cancer cases and deaths attributable to potentially modifiable risk factors in the United States. CA Cancer J Clin 2018;68:31–54.

doi: 10.3322/caac.21440.

[13] Wilson LF, Antonsson A, Green AC, Jordan SJ, Kendall BJ, Nagle CM, et al. How many cancer cases and deaths are potentially preventable? Estimates for Australia in 2013. Int J Cancer 2018;142:691–701. doi: 10.1002/ijc.31088.

[14] Care Information Centre S. HSE 2010 Chapter 10 Adult anthropometric measures, overweight and obesity 2010.

[15] Baracos VE, Martin L, Korc M, Guttridge DC, Fearon KCH. Cancer-associated cachexia. Nat Rev Dis Prim 2018;4:17105-. doi: 10.1038/nrdp.2017.105.

[16] Broder MS, Ailawadhi S, Beltran H, Blakely LJ, Budd GT, Carr L, et al. Estimates of stage-specific preclinical sojourn time across 21 cancer types. J Clin Oncol 2021;39:e18584–e18584. doi: 10.1200/jco.2021.39.15_suppl.e18584.

[17] Ferguson J, O'Connell M. Estimating and displaying population attributable fractions using the R package: graphPAF. Eur J Epidemiol 2024;39:715. doi: 10.1007/s10654-024-01129-1.

[18] Health Survey for England - 2011, Trend tables - NHS England Digital n.d. https://digital.nhs.uk/data-and-information/publications/statistical/health-survey-for-england/health-survey-for-england-2011-trend-tables (accessed 21 March 2026).

[19] Ovarian cancer statistics | Cancer Research UK n.d. https://www.cancerresearchuk.org/health-professional/cancer-statistics/statistics-by-cancer-type/ovarian-cancer (accessed 27 March 2026).
